# Dry loop-mediated isothermal amplification assay for detection of SARS-CoV-2 from clinical specimens

**DOI:** 10.20407/fmj.2022-003

**Published:** 2022-07-22

**Authors:** Yuki Higashimoto, Masaru Ihira, Yoshiki Kawamura, Masato Inaba, Kazuya Shirato, Tadaki Suzuki, Hideki Hasegawa, Tsutomu Kageyama, Yohei Doi, Tadayoshi Hata, Tetsushi Yoshikawa

**Affiliations:** 1 Faculty of Medical Technology, Fujita Health University, School of Medical Sciences, Toyoake, Aichi, Japan; 2 Faculty of Clinical Engineering, Fujita Health University, School of Medical Sciences, Toyoake, Aichi, Japan; 3 Department of Pediatrics, Fujita Health University, School of Medicine, Toyoake, Aichi, Japan; 4 Department of Infectious Diseases, Fujita Health University, School of Medicine, Toyoake, Aichi, Japan; 5 Department of Virology III, National Institute of Infectious Diseases, Musashimurayama, Tokyo, Japan; 6 Department of Pathology, National Institute of Infectious Diseases, Musashimurayama, Tokyo, Japan; 7 Influenza Virus Research Center, National Institute of Infectious Diseases, Musashimurayama, Tokyo, Japan; 8 Department of Clinical Laboratory, Fujita Health University Hospital, Toyoake, Aichi, Japan

**Keywords:** LAMP, SARS-CoV-2, COVID-19, Real-time RT-PCR

## Abstract

**Objectives:**

To establish a point-of-care test for coronavirus disease 2019 (COVID-19), we developed a dry loop-mediated isothermal amplification (LAMP) method to detect severe acute respiratory syndrome coronavirus 2 (SARS-CoV-2) RNA.

**Methods:**

We carried out reverse transcription (RT)-LAMP using the Loopamp SARS-CoV-2 Detection kit (Eiken Chemical, Tokyo, Japan). The entire mixture, except for the primers, is dried and immobilized inside the tube lid.

**Results:**

To determine the specificity of the kit, 22 viruses associated with respiratory infections, including SARS-CoV-2, were tested. The sensitivity of this assay, determined by either a real-time turbidity assay or colorimetric change of the reaction mixture, as evaluated by the naked eye or under illumination with ultraviolet light, was 10 copies/reaction. No LAMP product was detected in reactions performed with RNA from any pathogens other than SARS-CoV-2. After completing an initial validation analysis, we analyzed 24 nasopharyngeal swab specimens collected from patients suspected to have COVID-19. Of the 24 samples, 19 (79.2%) were determined by real-time RT-PCR analysis as being positive for SARS-CoV-2 RNA. Using the Loopamp SARS-CoV-2 Detection kit, we detected SARS-CoV-2 RNA in 15 (62.5%) of the 24 samples. Thus, the sensitivity, specificity, positive predictive value, and negative predictive values of the Loopamp 2019-CoV-2 detection reagent kit were 78.9%, 100%, 100%, and 55.6%, respectively.

**Conclusions:**

The dry LAMP method for detecting SARS-CoV-2 RNA is fast and easy to use, and its reagents can be stored at 4°C, solving the cold chain problem; thus, it represents a promising tool for COVID-19 diagnosis in developing countries.

## Introduction

The outbreak of coronavirus disease 2019 (COVID-19), caused by the novel coronavirus designated as severe acute respiratory syndrome coronavirus 2 (SARS-CoV-2), started in Wuhan, China, in December 2019.^1,2^ To date, the outbreak has spread rapidly around the world,^1,2^ and World Health Organization (WHO) has declared it a pandemic. On 3 March 2022, WHO reported a total of 437,333,859 confirmed COVID-19 cases and 5,960,972 associated deaths. Since SARS CoV-2 was first detected, the original virus has mutated, yielding an impressive array of variants. The accumulation of mutations arising out of subsequent viral replication is a natural phenomenon. WHO defines a variant of interest (VOI) as a variant with genetic changes impacting pathogen transmissibility and disease severity, as well as community transmission in multiple countries. A variant of concern (VOC) shares the characteristics of a VOI but is additionally associated with increased virulence and decreased effectiveness of public health measures. As of November 24, 2021, the five SARS-CoV-2 variants that have been designated as VOC are Alpha (B.1.1.7), Beta (B.1.351), Gamma (P.1), Delta (B.1.617.2), and Omicron (B.1.1.529).

Clinical trials have evaluated several drugs, with remdesivir and systemic corticosteroids showing promise for treating moderate and severe cases of COVID-19, respectively.^3^ Several COVID-19 vaccines have been authorized; however, COVID-19 vaccine coverage remains insufficient. Antiviral and neutralizing antibody therapies that reduce the risk of COVID-19 progression are needed. New treatments have recently been approved, such as Molnupiravir, nirmatrelvir plus ritonavir (an orally administered antiviral agent targeting SARS-CoV-2),^4,5^ casirivimab and imdevimab (a combination of two neutralizing monoclonal antibodies [REGEN-COV]),^6,7^ and sotrovimab (an engineered human monoclonal antibody that neutralizes SARS-CoV-2).^8^ Several characteristic clinical findings, such as fever and dry cough with typical signs of pneumonia on chest computed tomography,^9–11^ history of close contact with a patient who has COVID-19 or of visiting a COVID-19-endemic area,^12,13^ are useful in diagnosing COVID-19. However, many patients have mild symptoms or are asymptomatic, hampering the ability to make an accurate diagnosis on the basis of only clinical features.^14–16^ Therefore, a rapid diagnostic method is necessary to provide a definitive diagnosis of COVID-19.

Real-time reverse transcription-polymerase chain reaction (RT-PCR) is widely used for the diagnosis of COVID-19.^17,18^ This method is very useful for testing large numbers of samples at large hospitals, diagnostic companies, and local health facilities. However, a point-of-care (POC) test for COVID-19 is also important for use in the management of patients with suspected cases of COVID-19 in low-resource settings. Additionally, because the expansion of COVID-19 to developing countries is a major public health concern, there is an urgent need to develop rapid diagnostic tests for COVID-19. Performing real-time RT-PCR requires a special thermal cycler with precision optics that monitor fluorescence emission from sample wells. In contrast, the loop-mediated isothermal amplification (LAMP) method can amplify template nucleotides under isothermal conditions with efficiency and specificity as high as those of nested double PCR.^19^ Owing to its speed, ease of use, and cost-effectiveness, LAMP has been widely used for POC testing for various infectious diseases, including COVID-19.^19–22^ Furthermore, a dry LAMP reagent mixture, which can be stored at 4°C, is much easier to handle and more heat-stable compared with mixtures using liquid reagents. A dry LAMP reagent kits for the diagnosis of *Mycobacterium tuberculosis*,^23^ human African trypanosomiasis,^24^ malaria,^25^ and influenza (type A and H1 pdm 2009) have been commercially produced.^26^ These dry LAMP methods did not require the enzyme or reaction buffer to be aliquoted. Therefore, the dry LAMP method would be very useful for the diagnosis of tropical infectious diseases in regions including Africa,^27^ which is expected to be the next epicenter of COVID-19.

The aim of this study was to evaluate the performance of the LAMP method using dry reagents for the rapid diagnosis of COVID-19 infection. Although several studies on the use of LAMP to detect SARS-CoV-2 have already been published,^28–30^ this is the first study to evaluate the performance of dry LAMP reagents for this purpose.

## Methods

### Viruses and RNA for initial validation analysis

A total of 22 respiratory pathogens were used in our initial validation analysis of the specificity of the study primers. The strain names of these 22 pathogens are listed in Table 1. The Middle East respiratory syndrome coronavirus (MERS-CoV) EMC strain was kindly provided by Dr. Ron A. M. Fouchier, Erasmus Medical Center, Rotterdam, the Netherlands. The SARS-CoV Frankfurt 1 strain was kindly provided by J. Ziebuhr, University of Würzburg, Germany. The clinical isolates of human coronaviruses (HCoV)-HKU1, -OC43, -NL63, and -229E were described by previous studies.^31–33^ *In vitro*-transcribed RNA (GenBank accession number MN908947), synthesized using a ScriptMax Thermo T7 Transcription Kit (TOYOBO, Osaka, Japan), was used to determine the detection limit of the Loopamp SARS-CoV-2 Detection kit (Eiken Chemical, Tokyo, Japan).

### Clinical specimens

From March 7 to April 30, 2020, when the Wuhan strain of SARS-CoV-2 was prevalent, nasopharyngeal swabs were collected from patients suspected to have COVID-19 at Fujita Health University. Swab samples were collected using a flocked sterile plastic swab applicator and placed directly into 3 mL of BD universal viral transport medium (Becton, Dickinson and Company, Franklin Lakes, NJ, USA). RNA was extracted from the swab samples immediately. This study was approved by the institutional review board of Fujita Health University (No. HM19-493). Written informed consent was obtained from each patient.

### RNA extraction

Viral RNA was extracted from 140 μL of BD universal viral transport medium into which a nasopharyngeal swab had been immersed. RNA extraction was performed using the QIAamp Viral RNA mini kit (#52904, QIAGEN, Chatsworth, CA, USA) in accordance with the manufacturer’s instructions. After extraction, RNA was eluted in 60 μL of buffer AVE and stored at –80°C.

### Real-time RT-PCR

Real-time RT-PCR assays for detecting SARS-CoV-2 were performed using TaqMan Fast Virus 1-Step Master Mix (#4444436, Thermo Fisher Scientific, Waltham, MA, USA) in accordance with the manufacturer’s instructions. This master mix can perform RT and PCR all in one reaction well. The following primers and probes were used: NIID_2019-nCOV_N_F2, 5'-AAATTTTGGGGACCAGGAAC-3'; NIID_2019-nCOV_N_R2, 5'-TGGCAGCTGTGTAGGTCAAC-3'; and NIID_2019-nCOV_N_P2, 5'-FAM ATGTCGCGCATTGGCATGGA BHQ-3'.^34^ Single-well denaturation, reverse transcription, and amplification steps were performed on a QuantStudio 1 Real-Time PCR System (Thermo Fisher Scientific, Waltham, MA, USA) in the standard mode. Primer and probe concentrations were as follows: NIID_2019-nCOV_N_F2, 500 nM; NIID_2019-nCOV_N_R2, 700 nM; and NIID_2019-nCOV_N_P2, 200 nM. PCR conditions were as follows: reverse transcription at 50°C for 5 min; enzyme activation at 95°C for 20 s; and 45 cycles of denaturation at 95°C for 15 s and primer annealing/extension/fluorescence emission at 60°C for 60 s. The real-time RT-PCR reaction mixture (total volume, 20 μL) contained 5.0 μL of 4× Fast Virus Master Mix, 1.0 μL of primer–probe pre-mix, 5.0 μL of template RNA, and nuclease-free water.

### Reverse Transcription-LAMP

Reverse transcription (RT)-LAMP was carried out using the Loopamp SARS-CoV-2 Detection kit (#LMP403, Eiken Chemical, Tokyo, Japan) in accordance with the manufacturer’s instructions. The RT-LAMP method can synthesize cDNA from template RNA and apply LAMP technology to amplify and detect the cDNA with one step in a single tube. Primers were designed to target the N gene and RNA-dependent RNA polymerase (RdRp) gene. Because the entire mixture, except for the primers, is dried and immobilized inside the tube lid, 10 μL of purified RNA and 15 μL of SARS-CoV-2-specific primer sets were added to the bottom of the tube, after which the tube was inverted several times to resuspend the enzyme and buffer. The full reaction mixture was then collected at the bottom of the tube by performing a quick centrifugation. The mixture was subsequently incubated in a real-time turbidimeter (LA-200; Eiken Chemical Tokyo, Japan) for 35 min at 62.5°C. In LAMP, a large amount of DNA is synthesized, yielding a large amount of pyrophosphate ion byproduct. Pyrophosphate ions combine with divalent metallic ions to form an insoluble salt. Adding manganous ion and calcein, a fluorescent metal indicator, to the reaction solution allows the visualization of substantial alterations of the fluorescence during the one-step amplification reaction, which takes 30–60 min.^35^ For a visual evaluation of the level of fluorescence, the reaction tube was illuminated with an ultraviolet light by using an ultraviolet illumination system (WSE-5300; ATTO, Tokyo, Japan) and was also observed by the naked eye.

### Statistics analysis

To determine how the performance of the SARS-CoV-2 dry LAMP assay compared with that of real time RT-PCR assays for detecting SARS-CoV-2, a two-by-two table was created to calculate the sensitivity, specificity, positive predictive value, and negative predictive value of the Loopamp SARS-CoV-2 Detection kit. Analytical performance characteristics with 95% confidence intervals (CI) were calculated for the sensitivity, specificity, positive predictive value, and negative predictive value of the Loopamp SARS-CoV-2 Detection kit compared with the real-time RT-PCR analysis by using GraphPad Prism9 for Windows (GraphPad Software, San Diego, CA, USA).

## Results

### Specificity and sensitivity of the dry LAMP method for SARS-CoV-2 detection

**Figure 1:**
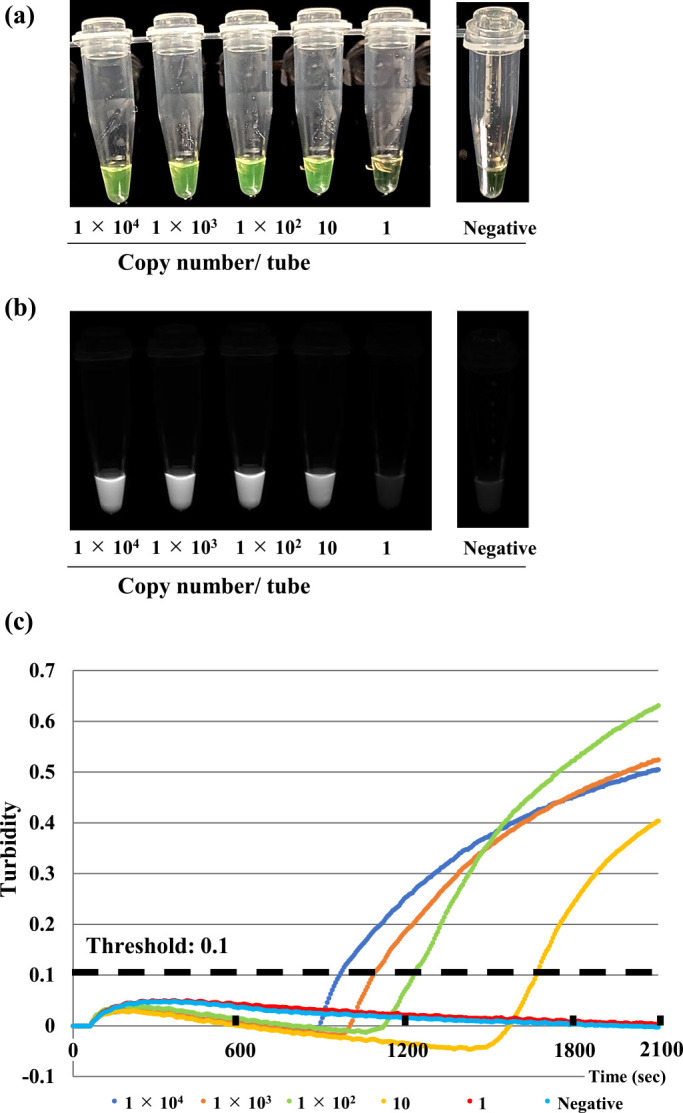
Sensitivity of the SARS-CoV-2 dry LAMP assay as determined using serially diluted *in vitro*-transcribed RNA. (a) Naked-eye detection at the end of the assay (35 min). Green indicates a positive reaction, and orange indicates a negative reaction. (b) Ultraviolet light detection at the end of the assay (35 min). Light gray indicates a positive reaction, and dark gray indicates a negative reaction. (C) Real-time turbidity assay.

To determine the specificity of the Loopamp SARS-CoV-2 Detection kit, we tested 22 viruses associated with respiratory infections, including SARS coronavirus, Middle East Respiratory Syndrome (MERS) coronavirus, other human coronaviruses, influenza viruses, and respiratory syncytial viruses. No LAMP product was detected in reactions performed with RNA from these pathogens (Table 1). These results were confirmed by a turbidity assay and agarose gel electrophoresis analysis (data not shown). To determine the sensitivity of the Loopamp SARS-CoV-2 Detection kit, *in vitro*-transcribed RNAs were serially diluted in 10 mM Tris buffer containing 0.1 mM EDTA, and 50 ng/mL of carrier RNA were used for defining the detection limit. The sensitivity of this assay, as determined by either the turbidity assay or a colorimetric change of the reaction mixtures evaluated by the naked eye, was 10 copies/reaction (Figure 1).

### Evaluation of the clinical application of the dry LAMP method for SARS-CoV-2 detection

To evaluate the performance of the Loopamp SARS-CoV-2 Detection kit as a POC test, we analyzed 24 nasopharyngeal specimens collected from patients suspected of having COVID-19, including three asymptomatic individuals who came into close contact with COVID-19 cases (Table 2). Of the 24 samples, 19 (78.9%) were determined to be positive for SARS-CoV-2 RNA when assessed by a real-time RT-PCR analysis. When using the Loopamp SARS-CoV-2 Detection kit, SARS-CoV-2 RNA was detected in 15 (62.5%) of the 24 samples. The four false-negative samples contained low copy numbers of viral RNA (2.6, 2.3, 2.2, and 0.5 copies/reaction) and were collected during the convalescent phase of illness (days 7 to 20). Thus, the sensitivity, specificity, positive predictive value, and negative predictive value of the Loopamp SARS-CoV-2 Detection kit were 78.9% (95% CI: 56.7%–91.5%), 100% (95% CI: 56.6%–100%), 100% (95% CI: 79.6%–100%), and 55.6% (95% CI: 26.7%–81.1%), respectively (Figure 2).

## Discussion

The LAMP assay has several advantages as a POC test method, such as its ease of use, speed, and low cost for amplifying target nucleotides. Accordingly, many investigators have developed SARS-CoV-2 LAMP assays.^21,28–30,36^ Several improvements to further leverage the advantages of the LAMP method have been also reported, including colorimetric detection using the naked eye^29^ or a fluorescent detector^36^ and direct detection of the target sequence without RNA extraction. These improvements shorten and simplify the workflow of the LAMP method^29^ and enable high-throughput analysis to be conducted. Performing the LAMP method with dry reagents will allow the use of this assay in developing countries because the dry reagents can be stored in refrigerators, which overcomes the requirement of traditional LAMP reagents for strict cold-chain transportation and storage. Therefore, in this study, we investigated the performance of the SARS-CoV-2 dry LAMP method.

No amplification was observed with RNA from other viruses (including SARS coronavirus, MARS coronavirus, and other human coronaviruses associated with respiratory infections) using the SARS-CoV-2 dry LAMP method (Table 1), indicating that this LAMP assay can specifically amplify SARS-CoV-2 RNA. In addition, the detection limit of the kit, based on both the turbidity assay and colorimetric change determined by the naked eye, was 10 copies/reaction, which is almost the same as or slightly higher than that of the previously reported SARS-CoV-2 LAMP assays.^36^ This initial validation analysis demonstrated that the SARS-CoV-2 dry LAMP method is highly specific and sensitive. Huang et al. showed that SARS-CoV-2 RNA can be amplified by a LAMP assay without RNA extraction,^29^ which is in line with findings from our previous studies for the detection of herpes simplex virus^37^ and human herpesvirus-6.^38^ Therefore, to further improve the LAMP method for use as a POC test, a direct detection method should be developed.

To evaluate the performance of SARS-CoV-2 dry LAMP when analyzing clinical samples, we tested 24 nasopharyngeal swab specimens collected from patients suspected of having COVID-19. The sensitivity and specificity of this assay was sufficient for practical purposes. In 24 nasopharyngeal swab specimens, there are only 5 SARS-CoV-2 RNA-negative samples. While there were four false-negative test results, as shown in Table 2, they were for the specimens with the lowest viral genome copy numbers. Notably, two of the four false-negative samples were collected from patients on days 18 and 20 post-symptom onset to confirm the absence of viral genome prior to hospital discharge. Because all samples with viral loads of more than 4.4 copies/reaction were accurately detected as SARS-CoV-2-positive by both turbidity assay and colorimetric changes, we believe that this SARS-CoV-2 dry LAMP method is sufficiently reliable for clinical use in diagnosing COVID-19.

In summary, this study demonstrated that the SARS-CoV-2 dry LAMP method is a reliable method for use in the rapid diagnosis of COVID-19. The dry LAMP method overcomes the requirement for strict cold-chain transportation and storage of reagents. Therefore, this method is expected to be a useful POC test in developing countries where COVID-19 is spreading.

## Figures and Tables

**Table1:** Specificity of dry loop-mediated isothermal amplification assay for SARS-CoV-2

Virus	Name of isolate		Amount (copies/reaction)
Coronaviruses			
	SARS-CoV	Frankfurt-1	4.00×10^7^
	MERS-CoV	EMC	1.00×10^5^

Human coronaviruses (HCoV)			
	HCoV-HKU1	Tokyo/SGH-15/2014	6.00×10^6^
		Tokyo/SGH-18/2016	1.00×10^6^
	HCoV-OC43	VR-1558	4.00×10^8^
		Tokyo/SGH-36/2014	3.00×10^6^
		Tokyo/SGH-61/2014	1.00×10^7^
		Tokyo/SGH-6/2015	4.00×10^6^
		Tokyo/SGH-65/2016	1.00×10^7^
	HCoV-NL63	Amsterdam I	8.00×10^7^
		Tokyo/SGH-15/2017	1.00×10^5^
		Tokyo/SGH-24/2018	4.00×10^4^
	HCoV-229E	VR-740	7.00×10^7^
		Sendai-H/1121/04	1.00×10^6^
		Niigata/01/08	1.00×10^5^

Influenza virus			
	A	A/Texas/50/2012(H3N2)	1.18×10^6^
		A/Narita/1/2009(H1N1)	2.94×10^6^
	B	B/Massachusetts/2/2012	4.44×10^6^
		B/Texas/2/2013	1.46×10^7^
		B/Brisbane/60/2008	2.00×10^5^

Respiratory syncytial virus (RSV)			
		A2	1.00×10^6^
		B1	1.00×10^6^

**Table2:** Comparison of real-time RT-PCR and dry loop mediated isothermal amplification assay results of SARS-CoV-2 using clinical specimens

Case	Sampling day*	Real-time RT-PCR (copies/reaction)	LAMP
1	unknown**	212732.5	+
2	unknown**	94749.3	+
3	7	22910.9	+
4	5	3796.9	+
5	11	1544.6	+
6	10	946.9	+
7	8	413.9	+
8	8	280.8	+
9	9	146.0	+
10	unknown**	68.7	+
11	12	49.8	+
12	12	48.9	+
13	7	14.2	+
14	8	13.2	+
15	19	4.4	+
16	7	2.6	−
17	18	2.3	−
18	10	2.2	−
19	20	0.5	−
20	14	0.0	−
21	8	0.0	−
22	12	0.0	−
23	10	0.0	−
24	12	0.0	−

* The day of disease onset was defined as day 1. ** Sampling day could not be determined because the patient was asymptomatic.

**Figure 2:** Diagnostic performance of the SARS-CoV-2 dry LAMP assay as compared with real time RT-PCR assays for detecting SARS-CoV-2. Abbreviations: +, positive, –, negative, PPV, positive predictive value, NPV, negative predictive value, CI, confidence interval.

	Real time RT-PCR		Sensitivity, % (95% CI)	Specificity, % (95% CI)	PPV, % (95% CI)	NPV, % (95% CI)
	+	−				
LAMP +	15	0	78.9 (56.7–91.5)	100 (56.6–100)	100 (79.6–100)	55.6 (26.7–81.1)
LAMP –	4	5				
